# ESR teleradiology survey: results

**DOI:** 10.1007/s13244-016-0485-6

**Published:** 2016-05-17

**Authors:** European Society of Radiology (ESR)

**Affiliations:** Neutorgasse 9/2, 1010 Vienna, Austria

**Keywords:** Teleradiology, PACS, Digital imaging, Imaging infomatics, Remote consultations

## Abstract

With recent developments of teleradiology technology and services, it has become necessary to better evaluate its extent and use among different countries in Europe. With this goal in mind, the ESR launched two specific surveys intended to gather the current state of adoption and implementation of teleradiology in clinical practice. A special focus on differentiating between insourcing teleradiology services among partners of the same organisation and outsourcing to external services was an essential part of the design of these surveys. The first survey was addressed to 44 national societies of different countries in Europe, while the second survey was intended for all practicing radiologist ESR members. While the results of these surveys reported here may provide a wealth of information to better understand the trends in adoption of teleradiology in Europe, they only represent a snapshot at a certain point in time. The rapid development of telecommunication tools as well as a fundamental change in practice and healthcare economics will certainly influence these observations in the upcoming years. These data, however, will provide objective and relevant parameters for supporting the efforts of experts and policy makers in promoting appropriate criteria and guidelines for adequate use of teleradiology in clinical practice.

*Main Messages*

• *Understand concepts and challenges of teleradiology*

• *Provide insight into current trends and solutions for teleradiology*

• *Compare differences in teleradiolgy strategies between countries in Europe*

• *Establish a reference on statistical data of usage of teleradiology in Europe*

## Introduction

The wider adoption of picture archiving and communication systems (PACS) has had significant benefits, including the ability to report on imaging procedures remotely, from the location where they have been performed via teleradiology. With the development of new technical infrastructures and networks allowing faster transfer of images and more accessible workstation designs, the rate of adoption of teleradiology in clinical practice has gained momentum in recent years. However, following publication of the White Paper on Teleradiology by the ESR in *Insights into Imaging* in 2014 [1], it has become apparent that the perspective of different National Societies varies, and in some countries there is a significant level of concern about how teleradiology is used currently, or might be used in the future. This particularly relates to outsourcing, and the effects that this might have on the quality and delivery of radiological services.

In order to further explore these differences and widen the scope of new applications of teleradiology and how they are progressively penetrating the market, the ESR eHealth & Informatics Subcommittee in cooperation with the ESR Quality, Safety and Standards Committee have elected to initiate two new surveys on this topic, one being destined for national societies and the other one destined for practicing radiologists and general members of ESR.

This paper reports the detailed results of these surveys with the aim to provide a global view of the situation of teleradiology in clinical practice in Europe at this point in time. Although it is certainly not comprehensive, it provides sufficient data for a better perception of the impact of different teleradiology strategies and the users opinion as well as the national societies’ positions on these emerging and often disruptive changes in practice. Furthermore, the survey sent to the ESR National Societies was designed to try to capture the perspective of National Society members of the ESR on these important issues to help inform the ESR and its future policy.

## Methods

The surveys were performed online and invitations to participate were sent by the ESR office to the ESR National Societies for the first one (referred as the “first” survey) and to the ESR members for the second one (referred as the “second” survey in this paper). Online questionnaires (SurveyMonkey Inc.) were designed and submitted as a web link allowing users to fill in their responses on any internet browser.

The survey submitted to the National societies consisted of 11 questions as well as a section for free text comments (see below in the results section for detailed listing of the questions). The questions were specifically focused on the different impact of insourcing versus outsourcing teleradiology services.

The second survey submitted to full member ESR radiologists consisted of an extended set of 34 questions divided into four sections:

- brief introduction and demographic questions
- information on the impact and user dimension of teleradiology services requested (e.g., in- and outsourcing, dis-/advantages, quality assurance, etc.)
- information on the financial aspects, informed consent as well as guidelines and legislation in the respective field
- evaluation of the survey itself and teleradiology in general

In addition to the specific questions, the survey also included a section of free text for comments and opinion responses.

The data were first extracted and summarised by ESR staff and reviewed by the chair of ESR eHealth & Informatics Subcommittee and the chair of the ESR Quality, Safety and Standards Committee before submission of the manuscript to the members of the eHealth & Informatics Subcommittee for review.

### Keywords and definitions used in the survey

Teleradiology: Transfer of radiological images and patient-related data between geographically different locations for the purposes of primary interpretation, expert consultation and/or clinical review by digital transmission. (ref. ESR white Paper on teleradiology)
Insourcing: Shared or Network reporting – transfer of images between sites to enable the radiologist to work offsite or report images from remote locations, but employment arrangements are unaffected and radiologists are paid by one of the institutions
Outsourcing: Worklists are outsourced to teleradiology companies, which employ radiologists (see more detailed definition in the ESR white Paper on teleradiology)

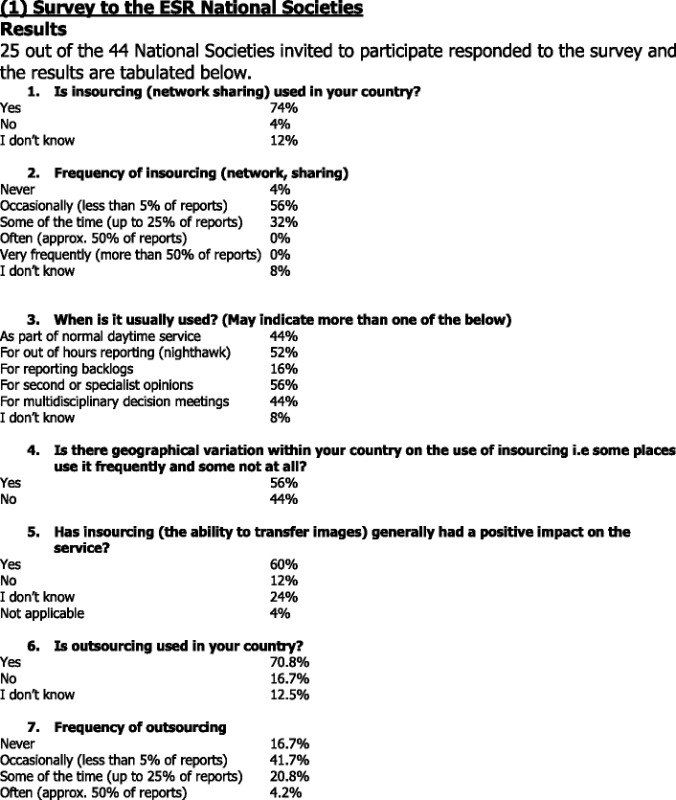

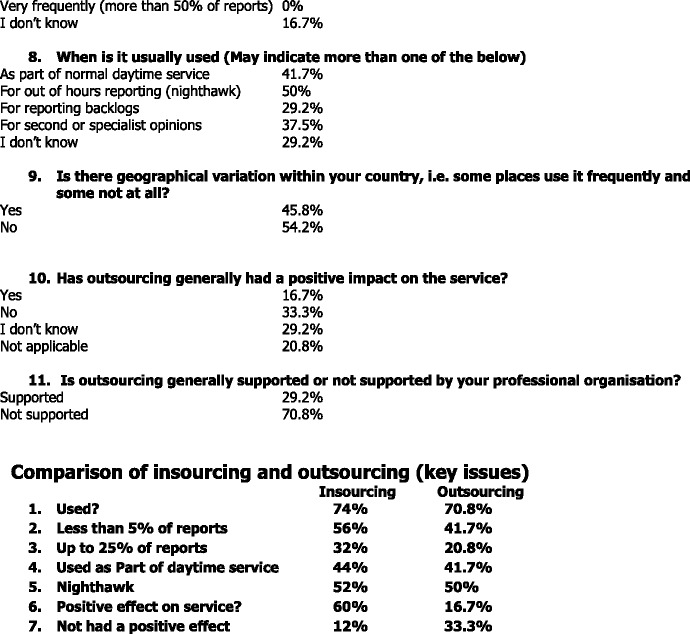


## Discussion

This survey mainly focused on differences in adoption of teleradiology and recommendations of different national societies regarding insourcing and outsourcing of services. However one of the key issues in discussing teleradiology is separating the provision of offsite services from the funding arrangements, and that is quite difficult to capture in a questionnaire. Also, various levels of service, from exam justification to interpretation and reporting may be provided offsite, although it is the remote interpretation and reporting that are most often provided via teleradiology. The general summaries of the survey are listed below.

### Insourcing

The majority of the respondents (74 %) answered that insourcing is used in their country, whereas 4 % answered that insourcing is not used; 12 % answered that they do not know. With regard to the frequency of insourcing (network, sharing), somewhat above half of the respondents (56 %) indicated that insourcing is used occasionally, i.e., in less than 5 % of the reports. In addition, 32 % answered that it is used some of the time, meaning in about up to 25 % of the reports. With respect to the purpose of using insourcing, the respondents could indicate more than one of the various answers offered. The majority (56 %) indicated that it is mostly used for second or specialist opinions and (52 %) for out of hours reporting – nighthawk. The same percentage (44 %) was indicated for multidisciplinary decision meetings and as part of normal daytime service.

In response to the question with regard to geographical variation within a country on the use of insourcing, i.e., some places use it frequently and some not at all; 56 % replied that there indeed is geographical variation, whereas 44 % answered that there is no variation. Free text comments indicated that the use of insourcing is dependent on the hospital organisation and structure. It was also indicated that it is usually used in distant (rural) facilities, smaller hospitals, and some private practices.

Furthermore, 60 % are of the opinion that insourcing (the ability to transfer images) generally had a positive impact on the service. Also, 12 % said that it did not have a positive impact, whereas 24 % answered that they do not know. The respondents could comment on the benefits and problems associated with insourcing (network or sharing). The societies indicated the possibility of receiving a second opinion and consultation in difficult cases and in facilities with limited experience, easier access to images and patient’s history, and bypassing the shortage of radiologists and thus managing the workload as the benefits of insourcing. Concerns about reducing the individual radiologist's productivity, the question of remuneration for insourcing, increasing workload in facilities with no specialists, and not perceiving radiologists as doctors were listed as disadvantages of insourcing.

### Outsourcing

For outsourcing, 70.8 % responded that outsourcing is used in their country, whilst in 16.7 % it is not used. Also, 12.5 % of the respondents indicated that they do not know. As regards the frequency of using outsourcing, 41.7 % use it occasionally, i.e., in less than 5 % of the reports and 20.8 % use it in up to 25 % of the reports. With regard to the purpose of using outsourcing, the respondents could again indicate more than one of the various answers offered. Outsourcing is mainly used for out of hours reporting – nighthawk (50 %). It is also used as part of normal daytime service (41.7 %), for second or specialist opinions (37.5 %), and for reporting backlogs (29.2 %).

As for the geographical variation within a country on the use of outsourcing, i.e., some places use it frequently and some not at all; 54.2 % answered that there is no variation, whereas 45.8 % said that there is variation. Free text comments indicated that the use of outsourcing is dependent on the network capacity of the hospitals, and that it is mainly used in smaller hospitals. Cross border outsourcing was not addressed in this survey.

Concerning the impact on the service, slightly above one third (33.3 %) indicated that outsourcing has no positive impact and only 16.7 % responded that outsourcing has a positive impact on the service. There was 29.2 % that answered with “I don’t know” and 20.8 % chose the answer “not applicable”.

The societies were solicited to comment on the benefits and potential problems associated with outsourcing. Regarding the benefits, respondents indicated the improved turnaround time and less workload, as well as being able to avoid a patient transfer with the help of an expert opinion on an imaging exam. The following are regarded as problems associated with outsourcing: lack of knowledge of the patient data and local circumstances; licensing, accreditation and legislation issues; the question of quality, and follow-up.

## Conclusions

The survey revealed that insourcing and outsourcing are used in many of the National Societies’ countries and their use is relatively evenly balanced, insourcing being used slightly more frequently than outsourcing. However when it comes to the positive impact on the service, it is perceived that insourcing mainly has a positive impact (60 %), whereas for outsourcing only 16.7 % perceived the impact to be positive. In 70.8 % of cases, the professional organisation stated that they did not in general support outsourcing. Despite positive effects on workload and quicker turnaround times, outsourcing in particular is associated with significant concerns such as quality, legal issues, and reducing the clinical role of radiologists.
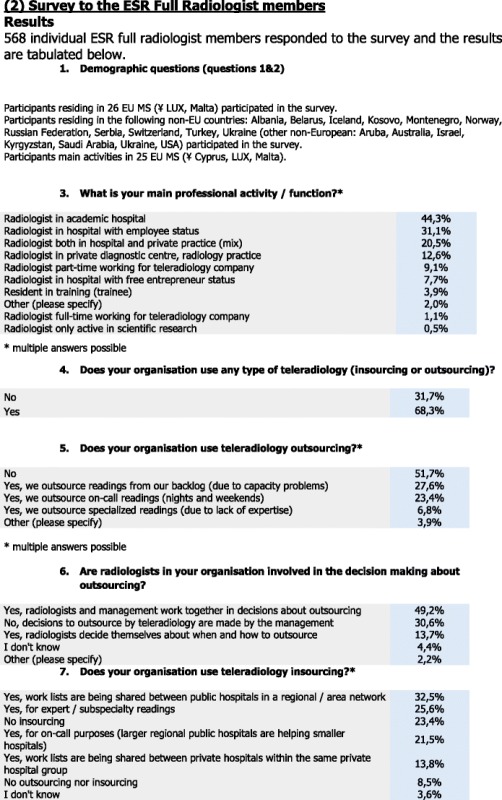

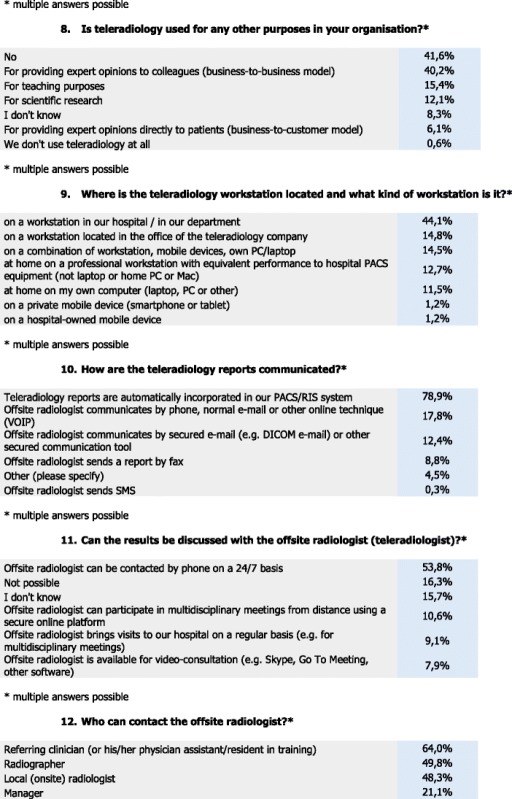

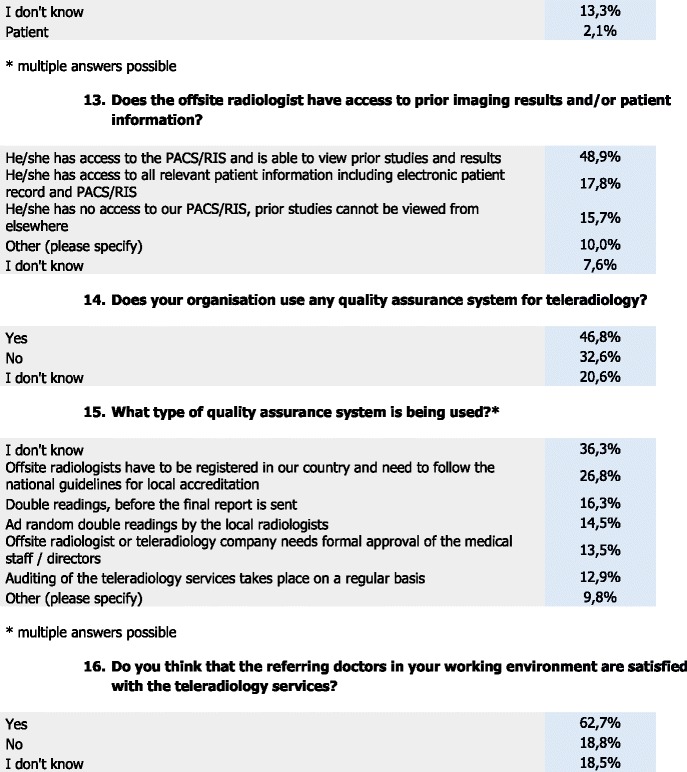

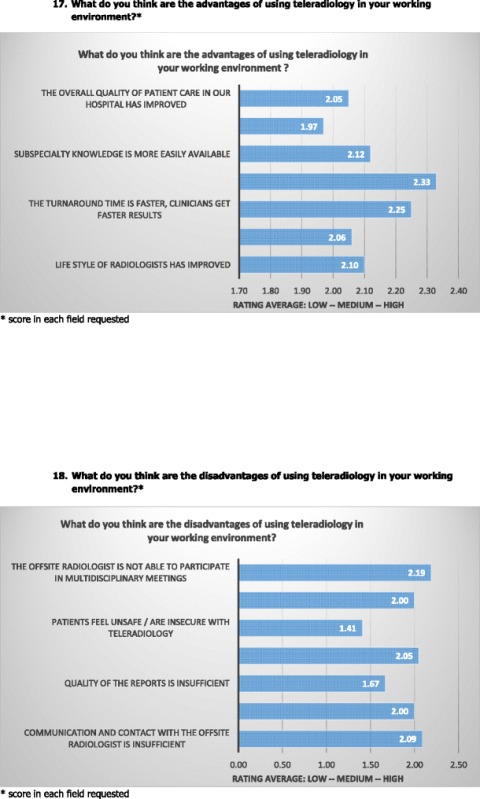

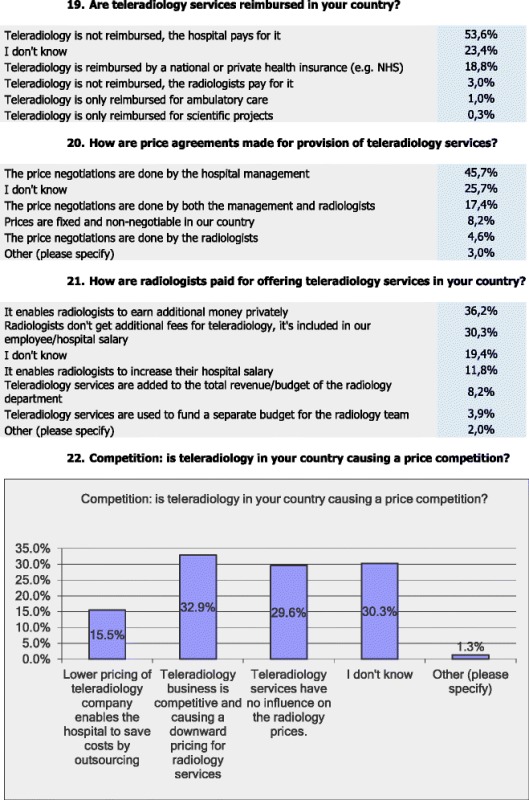

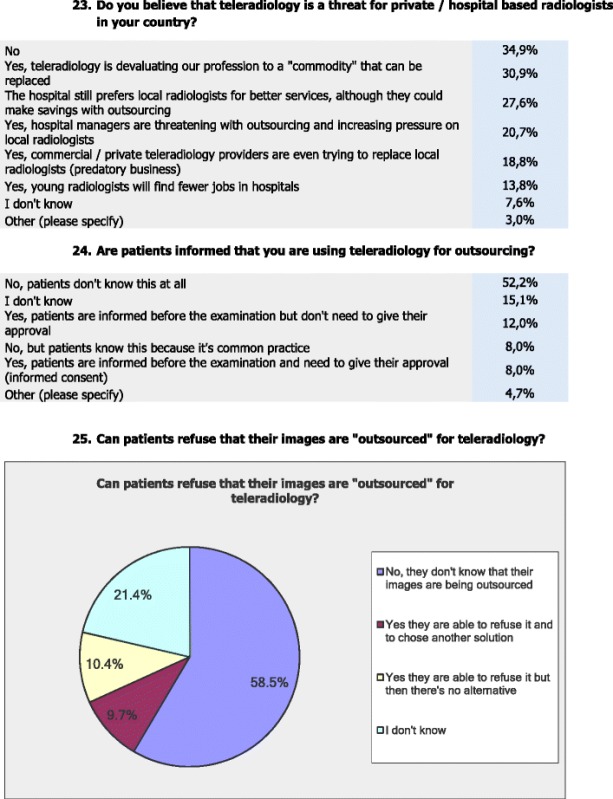

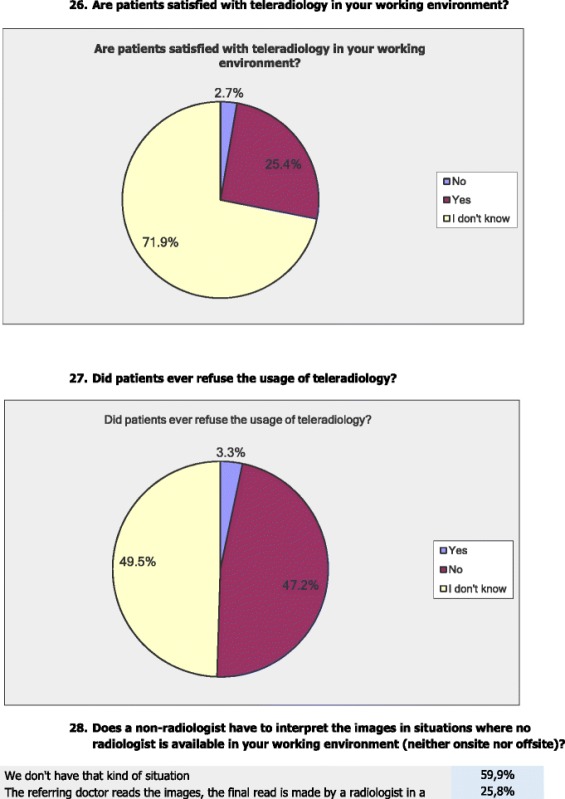

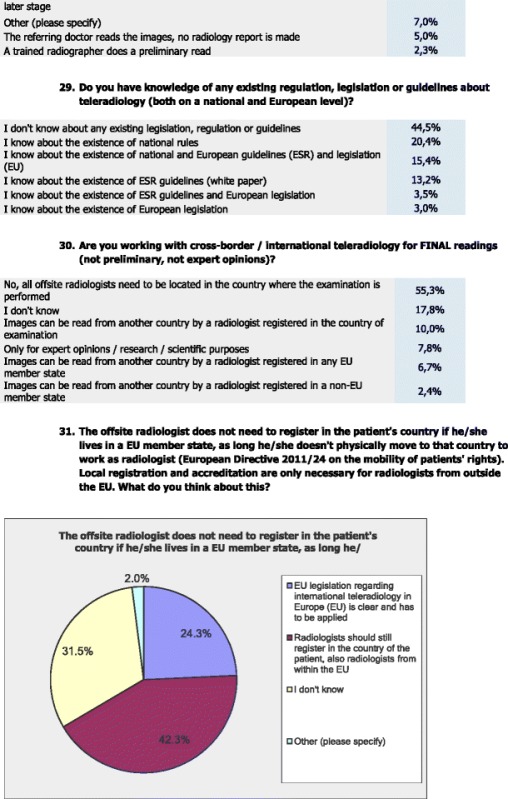


## Discussion

This second survey sent to ESR members is more extensive and covers a wide range of topics and issues. A summary of the specific answers to the questions regarding these different topics is listed below.

### Usage of teleradiology (TR)

The majority of the respondents (68.3 %) answered positively to the use of teleradiology (in- or outsourcing), whereas one third (31.7 %) answered that they did not use teleradiology (in- or outsourcing).

### Outsourcing

For outsourcing, 51.7 % stated that they did not use ‘TR outsourcing’, whereas 27.6 % indicated that ‘readings are outsourced from their backlog’, 23.4 % answered that ‘on-call readings are outsourced during nights and weekends’ and 6.8 % stated that ‘specialised readings are outsourced due to lack of experience’.

Almost half of the respondents (49.2 %) stated that both management and radiologists are involved in the decision-making process about outsourcing. One third (30.6 %) stated that solely the management decides on outsourcing. In addition, 13.7 % stated that the radiologists decide by themselves about when and how to outsource and about 4 % did not know about the decision-making process.

### Insourcing

More than one third (32.5 %) of the respondents stated that their organisation uses TR insourcing, whereas 25.6 % stated it was used for ‘expert/subspecialty readings’, 21.5 % stated it was used for on-call purposes, and 13.8 % stated that work lists are being shared between private hospitals within the same hospital group. Almost a quarter (23.4 %) of the respondents stated that they do not use insourcing, and 8.5 % stated that they do not use in- or outsourcing at all, while 3.6 % stated that they did not know about the procedure.

### Other purposes

There were 41.6 % that stated TR is not used for any other purposes, whereas 40.2 % responded that TR is used for providing expert opinions (business-to-business); 15.4 % stated that TR is used for teaching purposes, 12.1 % for scientific research, and 6,1 % for providing expert opinion directly to the patient. In addition, 8.3 % do not know about other purposes, and 0.6 % do not use TR at all.

### Location

For location, 44.1 % of the respondents stated that TR is used on a workstation in their hospital/department; 14.8 % stated that TR is used on a workstation located in the office of the TR company, 14.5 % responded that they use a combination of workstation, mobile device and own PC/laptop, and 1.2 % stated that they use a hospital-owned mobile device. Also, 12.7 % use TR at home on a professional workstation with equivalent performance to hospital PACS, 11.5 % use TR at home on their own computer, and 1.2 % use other private mobile devices (smartphone, tablet).

### Communication

The vast majority of respondents (78.9 %) stated that TR reports are automatically incorporated in their PACS/RIS systems, whereas 17.8 % stated that the offsite radiologists communicate by phone, normal email, or other online technique (VOIP). Also, 12.4 % stated that offsite radiologists communicate via a secured e-mail system (e.g., DICOM-e-mail protocol adopted as a national standard in Germany or other third party secure Email systems) and 8.8 % stated that the offsite radiologist sends a report by fax, while 4.5 % stated that they did not know about the communication protocols used and 0.3 % responded that the offsite radiologists sends an SMS.

Additionally, 53.8 % of the respondents stated that the offsite radiologist can be contacted by phone on a 24/7 basis, and 10.6 % stated that the offsite radiologist can participate in multidisciplinary meetings from a distance using a secure online platform, whereas 9.1 % stated that the offsite radiologist brings visits to their hospital on a regular basis, and 7.9 % stated that the offsite radiologist is available for video-consultations, e.g., via Skype, GTM, or other software. Also, 16.3 % responded that there is no possibility to discuss the results with the offsite radiologist at all and 15.7 % do not know.

Furthermore, 64 % of the respondents stated that the referring clinician can contact the offsite radiologist, whereas 49.8 % stated that the radiographer contacts the offsite radiologist, and 48.3 % stated that local (onsite) radiologist contacts the offsite radiologist. According to 21.1 % of the respondents, the management contacts the offsite radiologist, and 13.3 % do not know about the procedure. Also, 2.1 % stated that the patient can directly contact the offsite radiologist.

### Accessibility and quality assurance

For accessibility and quality assurance, 48.9 % of the respondents stated that the offsite radiologist has access to the PACS/RIS and, thus, is able to view prior studies and results, and 17.8 % stated that the offsite radiologist has access to all relevant patient information including electronic patient records and PACS/RIS. Also, 15.7 % responded that the offsite radiologist does not have access to PACS/RIS and prior studies cannot be viewed from elsewhere,and 7.6 % stated they do not know if offsite radiologists have access to prior imaging results and/or patient information.

Almost half (48.9 %) of the respondents stated that their organisation uses a quality assurance system for TR, whereas 32.6 % stated that they do not use a quality assurance system in their organisation for TR, and 20.6 % do not know whether their organisation uses quality assurance systems for TR.

Then 36.3 % of the respondents stated that they do not know what type of quality assurance systems are being used by their organisation for TR, whereas 26.8 % stated that offsite radiologists need to be registered in their country and need to follow the national guidelines for local accreditation, 16.3 % stated that double readings are made before sending the report and 14.5 % stated that random double readings are made by local radiologists. In addition, 13.5 % of the respondents stated that the offsite radiologist or teleradiology company needs formal approval of the medical staff/directors and 12.9 % stated that in their organisation the TR services are being audited on a regular basis and 9.8 % indicated other.

### Satisfaction, advantages & disadvantages of TR

More than two thirds (62.7 %) of the respondents stated that they think that the referring doctors are satisfied with the TR services within their working environment, whereas 18.5 % stated that they did not know and 18.8 % stated that they believe that the referring doctors are unsatisfied with the TR services.

The majority of respondents rated ‘greater availability of radiologists’ as the most important advantage of TR, second is ‘faster turnaround time, clinicians get faster results’, ‘more easily availability of subspecialty knowledge’ is in third place followed by ‘improvement of the radiologist’s lifestyle’, ‘improvement of local radiologist’s workload’ and ‘the improvement of the overall quality of care’. Whereas ‘the improvement of the overall quality of radiology services’ seems to be the least accurate advantage to the respondents of the survey.

As regards the disadvantages of TR usage, ‘the offsite radiologists unavailability to participate in multidisciplinary meetings’ seems to be the most important disadvantage for the respondents of the survey. This is followed by ‘insufficient communication and contact with the offsite radiologist’ in second place, ‘insufficient contact with the patient’ in third place and ‘the insufficient access of offsite radiologists to clinical and/or historical patient data’ and ‘insufficient contact between the offsite radiologist and radiographers’ in fourth place. ‘Insufficient quality of reports’ and ‘patients feel unsafe / are insecure with TR’ mark the least relevant disadvantages according to the respondents.

### Reimbursement, pricing, and competition

More than half (53.6 %) of the respondents stated that TR is not being reimbursed and that the hospital covers the costs, 23.4 % stated that they do not know whether TR is being reimbursed, 18.8 % stated that TR is reimbursed by a national or private health insurance, only 3 % stated that the radiologists pay for the TR services, 1 % stated that TR is only reimbursed in cases of ambulatory care and 0.3 % stated that TR is only reimbursed for scientific projects.

As regards the pricing arrangements, almost half (45.7 %) of the respondents stated that the hospital management is in charge of price negotiations. A quarter of the respondents (25.7 %) do not know who is in charge, 17.4 % stated that the negotiations are done by both management and radiologists, whereas 8.2 % stated that prices are fixed and non-negotiable in their country. Only 4.6 % of the respondents stated that the radiologists are in charge of the price negotiations and 3 % stated ‘other’.

According to 36.2 % of the respondents, radiologists earn additional private money through TR, whereas 30.3 % stated that radiologists do not get any additional payments through TR as it is included in the hospital/employee salary. Almost twenty percent (19.4 %) stated that they do not know how TR services are being paid for. More than ten percent (11.8 %) stated that TR increases their hospital salaries and 8.2 % stated that TR services are added to the total revenue/budget of the radiology dept. Only 3.9 % stated that TR services are used to fund a separate budget for the radiology team.

As regards the potential impact of TR on price competition, 32.9 % of the respondents stated that ‘TR is competitive and causing a downward pricing for radiology services’, whereas 29.6 % stated that ‘TR does not influence radiology prices’ and 30.3 % did not know about potential impacts on price competition. Also, 15.5 % stated that ‘lower pricing of TR companies enable hospitals to save costs via outsourcing’, and 1.3 stated ‘other’.

### Potential threat by TR for radiologists

Almost thirty-five percent (34.9 %) of the respondents stated that TR is no threat for private/hospital based radiologists, whereas 30.9 % stated that ‘TR devaluates the radiology profession to a “commodity” that can be replaced’ and 20.7 % stated that ‘hospital managers are threatening radiologists with outsourcing and increasing the pressure on local radiologists’. In addition, 18.8 % stated that ‘commercial/private TR providers are even trying to replace local radiologists’ and 13.8 % fear that ‘younger radiologists will find fewer jobs in hospitals through TR services’. Almost one third (27.6 %) believe that ‘hospitals still prefer local radiologists for better services, although they could make savings with outsourcing’ and 7.6 % do not know if TR is a possible threat to local onsite radiologists.

### Patient information and satisfaction

More than half (52.2 %) of the respondents stated that ‘patients do not know at all that TR is being used for outsourcing’ and 8 % stated that ‘the patients do not know that TR is being used for outsourcing, but the patients are aware that it is common practice to do so’, whereas 12 % stated that ‘patients are informed that TR services is being used for outsourcing but no approval by the patient is needed’ and 8 % stated that ‘informed consent by the patient is needed to use TR for outsourcing’, and 4.7 % stated ‘other’.

The vast majority of respondents (71.9 %) stated that they do not know if patients are satisfied with TR in their working environment, whereas 25.4 % stated that they believe that the patients are satisfied with TR and 2.7 % stated that they do not believe that patients are satisfied with TR.

Almost half (49.5 %) of the respondents stated that they do not know any cases where the patient refused TR, whereas 47.2 % stated that they never had the situation of patients refusing TR, and 3.3 % stated that they had the case where patients refused TR.

### TR readings by non-radiologists

Almost sixty percent (59.9 %) of the respondents stated that they never experienced the situation of a ‘non-radiologist’ having to interpret the images due to unavailability of onsite radiologists, whereas 25.8 % responded that the referring doctors read the images, and the final readings are made by a radiologist in a later stage. Also, 5 % stated that the referring doctors read the images and no radiology report is made and 2.3 % stated that the radiographer does a preliminary read.

### Cross-border/international TR and national/EU legislation

Almost forty-five percent (44.5 %) of the respondents are unaware of any existing legislative framework, regulations or guidelines, and 20.4 % stated that they do not know of any national rules in this regard. While 15.4 % stated that they are aware of the existence of national and European guidelines (ESR) and legislation (EU), whereas 13.2 % stated that they knew about the existence of the ESR guidelines (white paper) and only 3.5 % stated that they knew about ESR guidelines and EU legislation, and only 3 % stated that they knew about the existence of EU legislation.

More than half (55.3 %) of the respondents stated that all offsite radiologists need to be located in the country where the examination is performed and, thus, did not work with cross-border/international TR for FINAL readings (not preliminary or expert opinions). Also, 17.8 % do not know whether they worked with cross-border/international TR for FINAL readings (not preliminary or expert opinions). In addition, 10 % stated that images can be read from another country by a radiologist registered in the country of examination, 7.8 % stated that only for expert/research/scientific purposes, 6.7 % stated that images can be read from another country by a radiologists registered in any EU member state, and 2.4 % stated that indeed images can be read from another country by radiologists not registered in a non-EU member state.

As regards the EU legislation on the application of patients’ rights in the cross-border healthcare (Directive 2011/24/EU) regarding the registration of the offsite radiologist in the patient’s country, 42.3 % stated that they believe that the radiologist should still register in the country of the patient including radiologists working within the EU. Whereas 31.5 % responded ‘I don’t know’ and 24.3 % stated that the EU legislation regarding international TR in the European Union is clear to them and needs to be applied, but 2 % responded ‘other’.

## Conclusions

The results of this survey confirm a relatively wide adoption of teleradiology in Europe with a variable focus on what is the real application in clinical practice. However, the trend to use cross-border services and outsourcing for remote services remains limited. The lack of general regulations and legislation is of concern and the need for better quality control and some concerns about the level of adequate expertise. Differences between global EU legislation and local and national regulations may result in contradictions that can be confusing. The lack of awareness of the radiology community about these regulations may also be a source of inadequate apprehension of the fundamental guidelines in adopting teleradiology in clinical practice. Unawareness of the quality assurance (QA) methods in place or those that could be adopted was also striking from the fact that 36 % of the users stated that they did not know how QA was performed. It is, however, clear that the general reluctance toward use of teleradiology comes from fear of deteriorating hospital-based radiology and a commoditisation of low cost external services. It is also worth mentioning that these surveys showed that in a large majority of cases, patient opinion regarding TR was not known. Perhaps more attention should be paid to the patient’s point of view regarding usage of teleradiology, especially in the context of the current patient-centred trend in healthcare.

While the survey provides a wealth of resourceful data, a large range of comments and answers in free text listed in Appendix 1 may require more structured analysis to extract the general trends and opinions.

## Appendix 1

In order to access the full list of survey answers, please contact the *Insights into Imaging* editorial office at office@i3-journal.org

## References

[1] European Society of Radiology (2014). ESR white paper on teleradiology: an update from the teleradiology subgroup. Insights Imaging.

